# Fatal Fulminant Hepatitis from Rituximab-induced Hepatitis B Reactivation in a Patient with Follicular Lymphoma: A Case Report and a Brief Review of Literature

**DOI:** 10.7759/cureus.2257

**Published:** 2018-03-02

**Authors:** Zarak H Khan, Kamran Ilyas, Haider Ghazanfar, Hamza H Khan, Qulsoom Hussain, Sahla Hammad, Ahmed Munir, Rizwan Asim

**Affiliations:** 1 Department of Medicine, Shifa International Hospital, Islamabad, Pakistan; 2 Internal Medicine, Shifa College of Medicine, Islamabad, Pakistan; 3 Graduate, Shifa International Hospital, Islamabad, Pakistan; 4 Internal Medicine, Shifa International Hospital, Islamabad, Pakistan; 5 Department of Medicine, Services Hospital, Services Institute of Medical Sciences, Lahore, Pakistan; 6 Internal Medicine, Texas Tech University (perian Basin)

**Keywords:** hepatitis b, rituximab, follicular lymphoma, hematologic malignancies, viral hepatitis

## Abstract

The objective of our study was to recognize hepatitis B reactivation as a complication of rituximab chemotherapy and to realize the importance of screening for prior Hepatitis B virus (HBV) exposure in all patients with hematologic malignancies who will receive rituximab as part of their therapy. Rituximab is a monoclonal antibody targeting CD 20 receptors on the membrane of B cells. In this case report, we described a 79-year-old man who presented to our department with nausea, fatigue, and jaundice. Two months ago, he had received the last dose of the chemotherapy regimen containing rituximab for follicular B cell lymphoma. Ultrasound and computed tomography (CT) scan of abdomen did not show any focal lesions. Liver function tests showed worsening hepatic failure and viral serology demonstrated active HBV infection. Antiviral therapy with entecavir and tenofovir disoproxil fumarate failed to improve his symptoms, and he died of fulminant hepatic failure. Rituximab targets CD 20 receptors positive B cells. It can destroy both cancerous and normal B cells. A decline in immune function can activate occult HBV infection. Prior to initiation of rituximab therapy, screening should be conducted in all cases for HBV associated serological markers. Patients with active or occult HBV infection must be started on appropriate antiviral therapy to prevent any severe outcomes with rituximab-containing regimens.

## Introduction

In 1997, rituximab was approved for medical use and to treat non-Hodgkins B cell lymphomas. It is listed as one of the most effective and safest medicines in the health system. It binds to CD 20 receptors on B cells, leading to the destruction of B cells including both the normal and cancerous cells [1]. It is especially used to treat diseases which have a high number of B cells or are characterized by abnormal B cells. By eliminating abnormal B cells, it allows the body to make normal and healthy B cells. Normal B cells are rapidly depleted from the peripheral blood and remain depleted until nearly six months post-treatment, followed by a slow recovery [2]. In this case, we attribute the reactivation of hepatitis B virus (HBV)  to rituximab therapy which was started for follicular cell lymphoma. HBV reactivation leads to fulminant hepatic failure which caused the death of the patient. 

## Case presentation

A 79-year-old man, a known case of B cell lymphoma, presented with nausea, fatigue, and jaundice for the past two weeks. His symptoms have progressively worsened with time. The nausea is aggravated by the smell of food and gets relieved when he takes ondansetron. There is no history of fever and vomiting. He was previously treated with six cycles of cyclophosphamide, vincristine, prednisone, and rituximab chemotherapy, followed by a dose of rituximab as a maintenance therapy two months after the last cycle. His last dose of rituximab was two months ago. On physical examination, he was found to be hypotensive (blood pressure: 95/60) and had tachycardia (heart rate: 115 beats per minute). He was disoriented in time, place, and person and appeared extremely irritated. He was moderately dehydrated and had right upper quadrant tenderness. The patient was admitted to the intensive care unit (ICU) and started on intravenous (IV) fluid. The patient was started on strict hemodynamic monitoring in order to assess volume depletion and volume overload. Urine output was monitored in order to assess renal dysfunction. Gastrointestinal hemorrhage prophylaxis was started with proton pump inhibitor. There was no electrolyte abnormality. Liver function tests (LFTs), renal function test, and Hepatitis B serology were also done and are presented in Tables 1-3, respectively. 

**Table 1:** Liver function tests AST: Aspartate transaminase ALT: Alanine transaminase INR: International normalized tatio PTT: Partial thromboplastin time

Laboratory Tests	Value
Total bilirubin	29.3 mg/dl
Direct bilirubin	24.9 mg/dl
ALT	2122 U/L
AST	2013 U/L
Albumin	2.4 g/dl
Total protein	5.8 g/dl
INR	5.9
PTT	54.3 seconds

**Table 2:** Renal function tests BUN: Blood urea nitrogen

Laboratory Tests	Value
Creatinine	2.3 mg/dl
BUN	41 mg/dl

**Table 3:** Hepatitis B serology HBsAg: Hepatitis B surface antigen HBeAg: Hepatitis B early antigen HBV: Hepatitis B virus Anti HBs: Antibody to the hepatitis B surface antigen Anti HBe: Antibody to hepatitis B early antigen. Anti-HBc: Antibody to hepatitis B core antigen

Laboratory Tests	Value
HBsAg	Positive
HBeAg	Positive
HBV DNA	4530000 IU/ml
Anti HBs	Negative
Anti HBe	Negative
Anti HBc IgM	Negative

His liver enzymes were severely elevated, and he was found to have an active HBV infection. His creatine level was elevated. His previous creatinine value was normal. No prior hepatitis B serology was done before the start of the chemotherapy regimen. Computed tomography (CT) scan and ultrasound did not reveal any focal lesions. The patient was diagnosed with fulminant hepatic failure from rituximab-induced hepatitis B reactivation and was given antiviral therapy with entecavir and tenofovir disoproxil fumarat. Despite best efforts, he continued to deteriorate and died from fulminant hepatic failure.

## Discussion

Reactivation of hepatitis B can be a fatal complication following systemic chemotherapy or immunosuppressive therapy. Prior to initiation of immunosuppressive therapy, screening should be conducted in all cases for HBV-associated serological markers [3-4]. Reactivation occurs not only in hepatitis B surface antigen (HBsAg) positive patients, but also in HBsAg negative patients with antibody to hepatitis B core antigen  (anti-HBc) and antibody to the hepatitis B surface antigen (anti-HBs) positivity (occult infection) [5]. High HBV DNA viral load, male gender, absence of anti-HBs before chemotherapy, and rituximab and steroid combination therapy confer increased risk for reactivation [4, 6]. For HBsAg positive patients, antiviral therapy should be given at least for six months after completion of chemotherapy [7-8]. Patients with occult HBV infection (HBsAg negative, anti-HBc positive and undetectable DNA) should be followed closely by monitoring HBV DNA, alanine aminotransferase (ALT), and aspartate aminotransferase (AST) monthly during and after chemotherapy for at least one year, and antiviral therapy should be started upon detection of HBV DNA [9-10].

## Conclusions

All patients with hematologic malignancies must undergo hepatitis B screening prior to initiation of rituximab therapy. Those with positive serologic markers must undergo proper antiviral therapy and should be monitored closely. 
